# University of Buffalo

**Published:** 1855-10

**Authors:** 


### EDITORIAL DEPARTMENT.

Will soon be published, from the press of Messrs. Blanchard & Lea, of Philadelphia, "The Principles and Practice of Physical Exploration, as applied to the Diagnosis of Diseases of the Respiratory Organs. By Austin Flint, M. D."

*Octavo of from* 4 *to* 500 *pages.*

The well known ability of the writer is a sufficient guarantee that this work will be a valuable contribution to that department of medical research. We hope that it may meet with a warm reception among the author's old friends, the subscribers of the Buffalo Medical Journal.

---

*University of Buffalo.*—Before another number goes out to our readers, the term of instruction at this school will have commenced. From the spirit at present manifested by the faculty, it is evident that the coming season is to be marked by unusual diligence and activity in teaching. The unequaled clinical advantages of the school will be drawn upon to their utmost capacity, while thorough preparation is making for the comfort of students. The building, which, from its size and arrangement, it has always been somewhat difficult to warm, will—if there is any faith in furnacemen—be comfortably heated in all parts.

We hope that practitioners will recollect the arrangement for their benefit in the January lectures. We shall inclose circulars to all our subscribers in due time, giving the particulars.

Students wishing to dissect are requested to be present at the preliminary term, as during the regular term their time will be fully occupied with the daily lectures.

